# Formation of diffusion barrier layers for thermoelectric modules via electroless Ni–P plating

**DOI:** 10.3389/fchem.2026.1856378

**Published:** 2026-07-28

**Authors:** Kangeun Lee, Juno Lee, Dong Gyu Yook, Jae-Hong Lim

**Affiliations:** Department of Materials Science and Engineering, Gachon University, Seongnam, Gyeonggi-do, Republic of Korea

**Keywords:** diffusion barrier layer, electroless plating, Ni-P, sodium dodecyl sulfate, thermoelectric element, wettability

## Abstract

In this study, electroless Ni–P plating was applied to thermoelectric elements to create a diffusion barrier layer. To achieve selective Ni–P plating, a surface activation process involving sensitization and activation treatments was applied. This pre-treatment protocol promoted the formation of a uniform Ni–P layer and enabled selective plating through wettability differences, which were regulated using sodium dodecyl sulfate as an anionic surfactant. The deposited Ni–P layer functioned effectively as a diffusion barrier by suppressing the formation of intermetallic compounds between the solder and the thermoelectric elements. The influence of sodium dodecyl sulfate on surface chemistry and wettability was investigated using contact angle measurements and X-ray photoelectron spectroscopy. Additionally, the microstructure and interfacial characteristics of the Ni–P and Cu/Ni–P-plated layers were examined by electron probe microanalysis (EPMA). The EPMA results revealed that the Ni–P layer effectively suppressed Cu diffusion into the thermoelectric elements even after thermal treatment, demonstrating its potential as a reliable diffusion barrier for thermoelectric module applications.

## 1 Introduction

Due to their ability to convert thermal energy into electrical energy, thermoelectric (TE) elements are commonly employed in energy harvesting applications, such as waste heat recovery. For example, TE elements based on Bi–Te alloys are widely applied in TE cooling and low-temperature (<250 °C) power generation (Hwang et al., 2019).

Conventional TE ingot fabrication requires multiple processing steps, limiting TE fabrication yields to ∼41% (Hwang et al., 2019). In contrast, hot extrusion can be used to directly produce TE elements, minimizing processing steps, reducing kerf loss, and increasing yields. Furthermore, coating the cylindrical TE element sidewalls with a polymer layer can enable selective plating and facilitate large-scale production (Hwang et al., 2019).

When constructing a TE module, Cu-based interconnections are commonly required as electrical connections. However, under high-temperature conditions, Cu can diffuse into other metals to form intermetallic compounds (Bae et al., 2019; Lin et al., 2012; Nguyen et al., 2021). This can lead to reduced electrical conductivity and degradation of mechanical properties, which should ideally be prevented (Li et al., 2015; Xia et al., 2014). In TE devices, such degradation is particularly problematic since increased contact resistance at the metal/TE element interface can significantly reduce charge-carrier transport efficiency and hinder device performance (Li et al., 2015).

To prevent the formation of intermetallic compounds and preserve the electrical and mechanical stability of electronic and semiconductor devices under high-temperature operating conditions, diffusion barrier materials have been designed to effectively suppress atomic migration (Lin et al., 2011; Lin et al., 2012; Russell, 2003; Tan et al., 2019). On TE element substrates, diffusion barrier layers can be formed through both electrochemical and physical processes. Electrochemical methods include wet processes such as electroplating and electroless plating (Bae et al., 2019; Hsieh et al., 2017; Lin et al., 2012; Nguyen et al., 2021; Yusufu et al., 2020; Zhu et al., 2022), whereas physical methods include constrained spark plasma sintering and physical vapor deposition (Liu et al., 2025; Shen et al., 2023; Song et al., 2014).

Recent studies have expanded barrier-layer design for Bi_2_Te_3_-based TE devices to include electroless Co–P/Ni–P multilayer barriers and Ti-based barriers, highlighting the need to consider diffusion blocking, contact resistance, mechanical reliability, and thermal stability together (Shui et al., 2025; Sun et al., 2023). In this study, electroless Ni–P plating was selected as a practical wet-process approach for forming a Cu diffusion barrier layer on polymer-coated cylindrical TE elements because of its uniform coating capability and suitability for TE module integration.

Electrochemical approaches, including electroplating and electroless plating, offer several advantages, such as low processing temperature, simple equipment, and compatibility with large-scale wet processing. Electroless Ni–P plating, in particular, has been employed as a rapid, uniform, and cost-effective method for forming Ni–P diffusion barrier layers on various TE elements to prevent Cu diffusion (Molaei and Atapour, 2024; Tan et al., 2019). However, electroplating requires an externally applied current and can be affected by current-distribution-dependent nonuniformity, particularly for the small cylindrical and partially polymer-coated TE elements used in this study. Such current-distribution nonuniformity may lead to thickness variation, localized deposition, or undesired metal growth near the TE element/polymer boundary. In contrast, electroless plating proceeds through a surface-catalyzed chemical reduction reaction without external current, making it more suitable for forming a conformal Ni–P layer on geometrically complex TE elements. Nevertheless, electrochemically deposited barrier layers, including electroless deposits, can exhibit low crystallinity or partially amorphous structures, which may limit their diffusion suppression performance under long-term thermal exposure.

The use of electroless plating is also advantageous for evaluating the role of SDS, which is used in this study as a key additive for controlling the wetting behavior and deposition characteristics of the plating bath. In electroplating, the effect of SDS on deposit morphology can be coupled with current density, local electric-field distribution, and cathodic polarization, making it difficult to separate surfactant-induced wetting effects from electrochemical current effects. By contrast, in electroless plating, deposition is mainly governed by surface activation, solution wettability, and autocatalytic reduction at the solid–liquid interface. Therefore, electroless Ni–P plating provides a more direct platform for evaluating how SDS controls bath wettability, surface coverage, deposition uniformity, and selective plating between the TE element and polymer regions. Based on these considerations, electroless Ni–P plating was employed in this study to form a Cu diffusion barrier layer selectively on the exposed TE element surface of polymer-coated cylindrical TE elements, while minimizing current-distribution-related nonuniformity and clarifying the role of SDS in wettability-driven selective Ni–P deposition. This selective deposition is necessary because unwanted metal growth on the polymer region could introduce additional thermal conduction pathways and reduce the temperature gradient required for efficient TE operation.

To achieve selective plating while maintaining a uniform and defect-minimized diffusion barrier, it is essential to control both interfacial wetting and deposition stability. In this context, sodium dodecyl sulfate (SDS), an anionic surfactant, can be introduced as a simple bath additive to tailor the wetting behavior of the plating solution and stabilize the deposition interface. Specifically, SDS can significantly reduce the contact angle of the plating solution, thereby enhancing solution spreading and interfacial contact on the target surface during electroless plating (Ivanova and Starov, 2011). Previous studies have also reported that surfactant addition can refine the deposit structure and improve coating uniformity (Malfatti et al., 2007). Related electrochemical deposition studies have also indicated that SDS adsorption at the substrate or growing deposit surface suppresses abnormal grain growth and leads to grain refinement, together with a more uniform microstructure (Farzaneh et al., 2010). SDS-assisted electroless plating is therefore expected to simultaneously support wettability-driven selective deposition on hybrid TE element/polymer substrates and enhance the morphological quality of the Ni–P diffusion barrier layer.

Previous studies on diffusion barrier layers fabricated via wet electrochemical methods have mainly focused on the structural characteristics of the thin films and their effectiveness in suppressing elemental diffusion. For example, Yusufu et al. (2020) deposited Ni layers on TE devices via sputtering or electroplating, followed by Au sputtering to fabricate a diffusion barrier against Cu diffusion. Similarly, Hsieh et al. (2017) employed electroless plating to form a Co–P diffusion barrier layer on TE element substrates, effectively preventing elemental diffusion from the Ni-based solder paste. These studies primarily evaluated the diffusion barrier performance through elemental analysis and microstructural observations.

**FIGURE 1:**
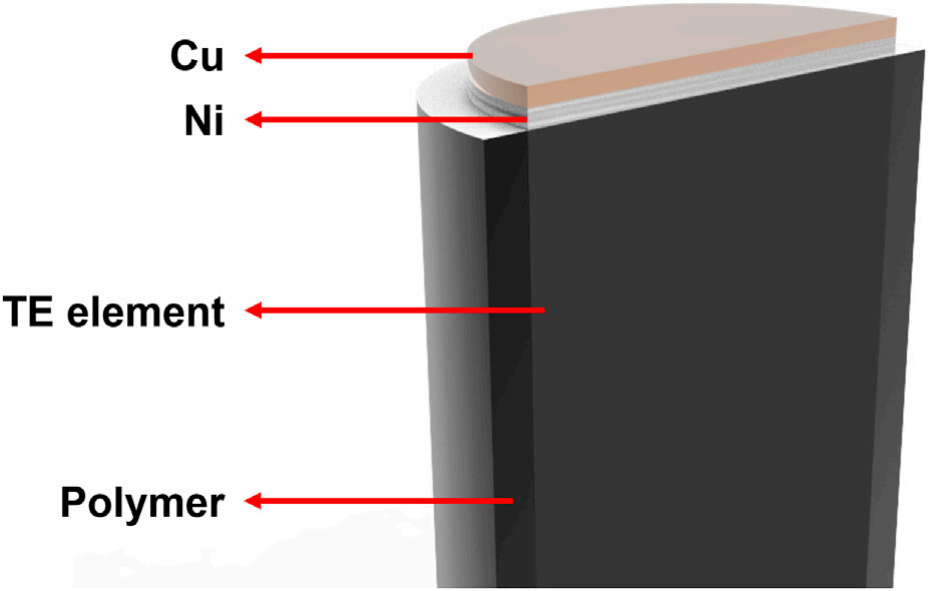
Structure of the electroless Ni–P-plated TE element.

With the above considerations in mind, this study adopts an electrochemical approach, specifically electroless Ni–P plating, to fabricate diffusion barrier layers for TE elements and evaluate their characteristics. To prevent Cu diffusion, electroless Ni–P plating is employed to form a Ni–P diffusion barrier layer on p-type Bi_2−x_Sb_x_Te_3_ (x = 1.56) TE elements (Figure 1). Cylindrical TE elements are directly fabricated via a hot extrusion process, and their sidewalls are coated with a polymer layer. In addition to evaluating the diffusion suppression performance of the Ni–P diffusion barrier layer, this study systematically investigates the physicochemical properties of the electroless Ni–P plating solution and the characteristics of the resulting Ni–P film as a function of surfactant addition. The novelty of this study lies in the integrated investigation of selective electroless Ni–P plating and the resulting surface and interfacial chemical changes directly on TE elements. Unlike previous studies that mainly focused on bath modification or diffusion barrier performance on conventional substrates, this work targets the formation of a Cu diffusion barrier layer for polymer-coated cylindrical TE elements. Furthermore, the effects of SDS addition on plating selectivity, the surface and interfacial chemical states of the Ni–P-plated TE elements, and Cu diffusion barrier performance are systematically examined, providing a more comprehensive understanding of diffusion barrier formation for TE module applications.

## 2 Materials and methods

**FIGURE 2:**
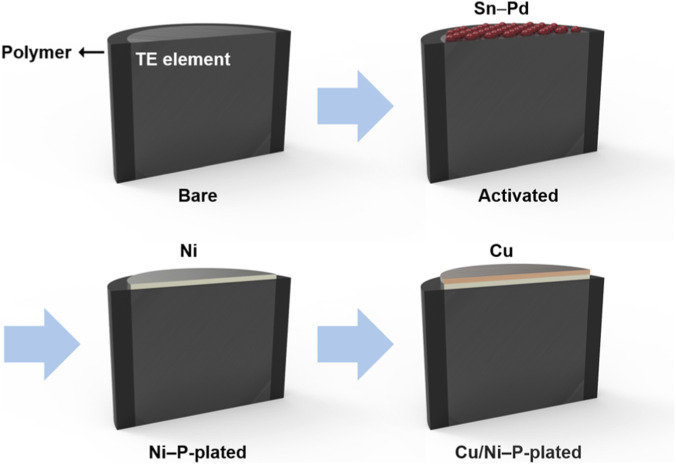
Outline of the experimental procedure for electroless Ni–P plating and Cu electroplating on the TE element.

As a general overview, the TE elements used in this study were fabricated by an extrusion process and sliced into cylindrical shapes (diameter = 1.3 mm, height = 5 mm). The side surfaces of the TE elements were encapsulated with polymer material, while the top and bottom surfaces were deliberately left exposed to facilitate the subsequent metallization and soldering processes. The encapsulating polymer was applied in the form of a spray-type paint containing 5% TiO_2_, 5% xylene, 5% toluene, and 5% 2-butoxyethanol. The polymer layer also served as an insulating layer to prevent short circuits during TE module assembly. Before plating, surface impurities were removed from the TE elements using an ultrasonic cleaner (WUC-A03H, DAIHAN, Korea). Each specimen was cleaned for 3 min each in acetone (99.5%, Samchun Chemicals, Korea), ethanol (99.9%, Samchun Chemicals, Korea), and deionized water (New Human RO-180, Human, Korea; water quality: 0–30 μS/cm, flow rate: max. 18 L/h). After cleaning, the specimens were dried at 70 °C in a convection oven (OF-02, Jeio Tech, Korea). The dried specimens were then subjected to electroless Ni–P plating to form a diffusion barrier layer, followed by Cu electroplating for electrical interconnection. Figure 2 provides a schematic diagram of the plating process.

Specifically, to activate the TE element surface for Ni‒P electroless plating, sensitization and activation processes were performed (Cui et al., 2011; Wei and Roper, 2014). The cleaned and dried TE elements were immersed in a sensitization solution containing 0.07 M SnCl_2_·2H_2_O (98%, Sigma-Aldrich, USA) and 0.3 M HCl (20% solution, Samchun Chemicals, Korea) for 3 min under sonication (WUC-A03H, DAIHAN, Korea), followed by rinsing with deionized water and drying at 70 °C in a convection oven (OF-02, Jeio Tech, Korea). Subsequently, the specimens were immersed in an activation solution containing 560 μM PdCl_2_ (≥99.9%, Sigma-Aldrich, USA) and 76.8 mM HCl (20% solution, Samchun Chemicals, Korea) for 3 min under sonication, then rinsed with deionized water and dried at 70 °C in a convection oven to complete surface activation.

The plating baths were prepared in deionized water and contained 35 g/L NiSO_4_·6H_2_O (98.5%–102.0%, Samchun Chemicals, Korea), 35 g/L NaH_2_PO_2_·H_2_O (≥99%, Sigma-Aldrich, Switzerland), 25 g/L Na_3_C_6_H_5_O_7_·2H_2_O (≥99%, Sigma-Aldrich, Canada), 30 g/L (NH_4_)_2_SO_4_ (≥99%, Sigma-Aldrich, Netherlands), and 2 ppm thiourea (CH_4_N_2_S, ≥99%, Sigma-Aldrich, India). To investigate the effect of adding a surfactant, Bath A was prepared without SDS while Bath B also contained 0.5 g/L SDS (NaC_12_H_25_SO_4_, ≥99%, Sigma-Aldrich, Japan). Electroless plating was performed at 80 °C for 20 min at pH 8.5 (adjusted using a 28.0%–30.0% ammonia solution, Samchun Chemicals, Korea).

Subsequently, Cu electroplating was carried out to form a Cu solder layer on the Ni–P diffusion barrier. The electrolyte consisted of 0.85 M CuSO_4_·5H_2_O (99.0%, Samchun Chemicals, Korea), 0.55 M H_2_SO_4_ (95%, Samchun Chemicals, Korea), 2 ppm polyethylene glycol (PEG, Mw ≈ 2000, Sigma-Aldrich, USA), and 500 ppm NaCl (99.0%, Samchun Chemicals, Korea). Cu electroplating was performed at a current density of −60 mA/cm^2^ for 8 min. A three-electrode system was employed, consisting of a Pt wire as the counter electrode, a Ni–P-plated TE element as the working electrode, and an Ag/AgCl electrode as the reference electrode, with an inter-electrode distance of 3 cm.

To evaluate the thermal stability of the diffusion barrier and investigate the extent of intermetallic compound formation, the Cu/Ni–P-plated TE elements were heat-treated at 100 °C and 200 °C under an argon atmosphere. The heat-treatment temperatures of 100 °C and 200 °C were selected to evaluate the thermal stability of the Ni–P barrier layer under conditions coincident with the peak-performance temperature range of p-type Bi_2_Te_3_-based TE materials (approximately 25 °C–125 °C) and under a relatively severe, thermally accelerated condition beyond this range, respectively. The heat treatment was performed under an Ar atmosphere to minimize oxidation of Cu, the Ni–P barrier layer, and the thermoelectric material, thereby allowing Cu diffusion behavior and interfacial stability to be examined more clearly. Additionally, to evaluate the effect of surfactant addition, contact angle measurements (Phoenix-10, SEO, Korea) and X-ray photoelectron spectroscopy (XPS, Axis Supra+, Kratos, UK) were employed. The uniformity and morphology of the Ni–P layer were analyzed using optical microscopy (OM, Hirox, MXB-5000REZ, Japan) and field-emission scanning electron microscopy (FE-SEM, S-4300, Hitachi, Japan). The presence of a catalytic Pd layer was confirmed using energy dispersive X-ray spectroscopy (EDX, EMAX 7021-H, HORIBA, Japan). The effectiveness of the Ni–P diffusion barrier in preventing intermetallic compound formation was further investigated by electron probe microanalysis (EPMA, JXA-8530F, JEOL, Japan) following heat treatment.

## 3 Results

**FIGURE 3:**
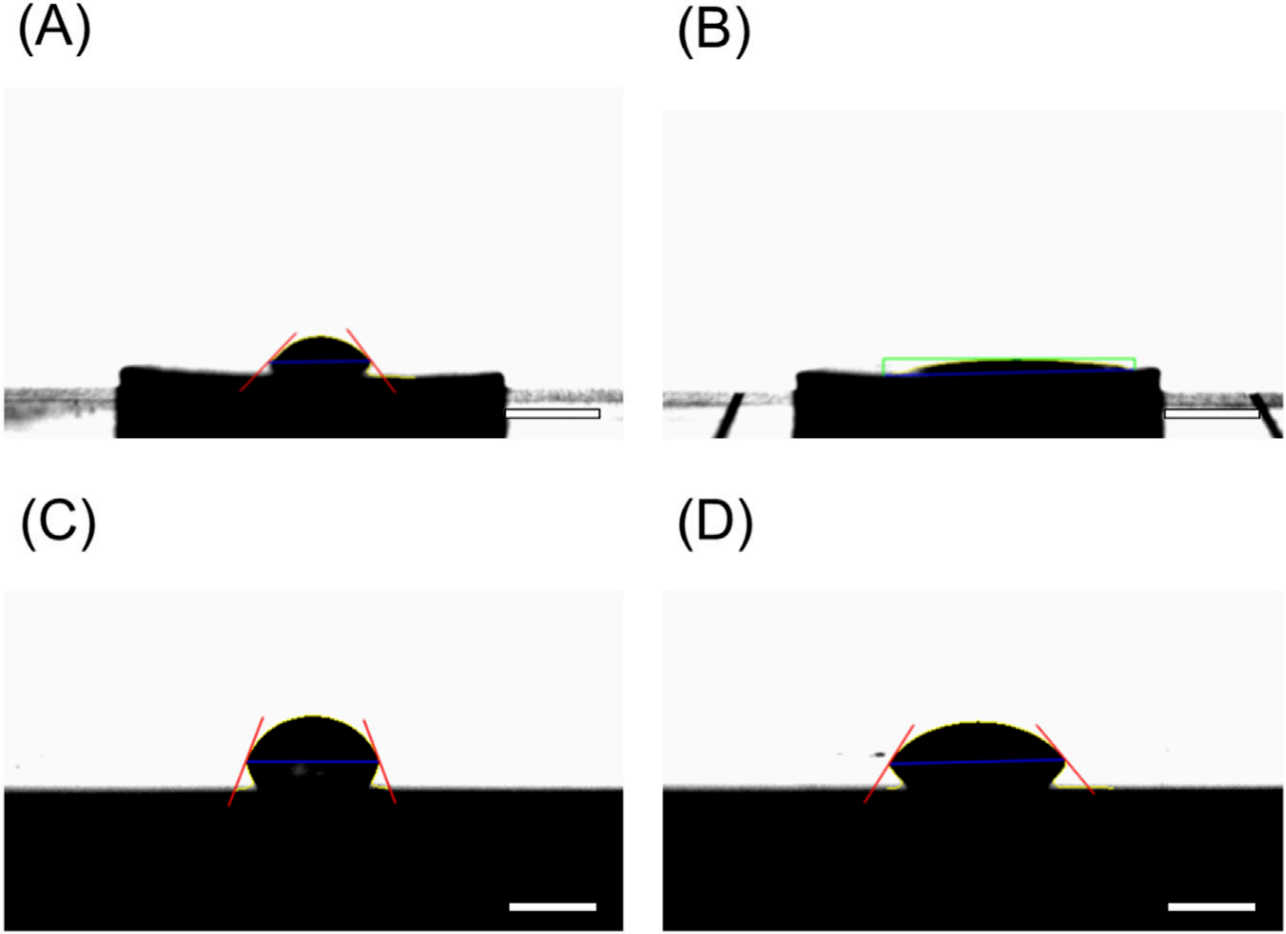
Contact angle measurements performed in the presence and absence of SDS in the electroless Ni–P plating bath: **(A)** TE element surface in Bath A (without SDS), **(B)** TE element surface in Bath B (with SDS), **(C)** polymer surface in Bath A (without SDS), and **(D)** polymer surface in Bath B (with SDS).

Contact angle analysis (Figure 3) revealed that the addition of SDS enhanced the wettability of the electroless Ni–P plating bath solution on the substrate. The contact angle of Bath A (without SDS) was 50° (Figure 3A), whereas a markedly reduced contact angle of 9° was measured for Bath B (with SDS, Figure 3B). In contrast, contact angle measurements performed between the electroless plating solution and the polymer surface revealed a contact angle of 73° in the absence of SDS (Bath A, Figure 3C) and a reduced contact angle of 56° with SDS addition (Bath B, Figure 3D), indicating that SDS also improves the wettability of the polymer surface. However, even with added SDS, the contact angle on the polymer surface remained relatively high compared with that on the TE element substrate.

**FIGURE 4:**
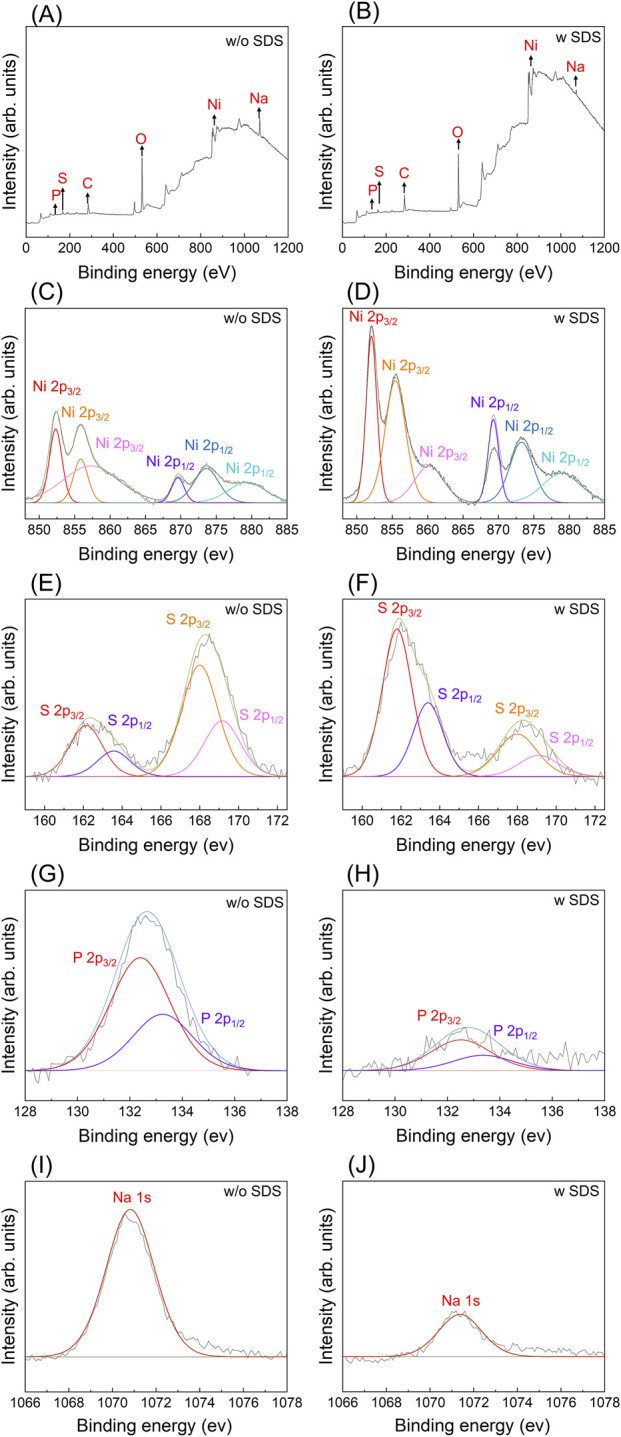
XPS spectra of electroless Ni–P-plated TE element surfaces prepared with and without SDS in the plating bath. Survey spectra acquired over the full binding-energy range for samples plated **(A)** without SDS and **(B)** with SDS. High-resolution Ni 2p spectra for samples plated **(C)** without SDS and **(D)** with SDS. High-resolution S 2p spectra for samples plated **(E)** without SDS and **(F)** with SDS. High-resolution P 2p spectra for samples plated **(G)** without SDS and **(H)** with SDS. High-resolution Na 1s spectra for samples plated **(I)** without SDS and **(J)** with SDS.

To investigate the effect of SDS addition on the chemical states of Na, S, Ni, and P on the Ni–P-plated surfaces, XPS was employed. Figures 4A,B show the survey XPS spectra recorded over the full binding-energy range for samples prepared in the absence and presence of SDS, respectively. The corresponding Ni 2p, S 2p, P 2p, and Na 1s spectra are shown in Figures 4C–J. The XPS spectra were analyzed using CasaXPS software. Binding energies were calibrated with respect to the C 1s peak at 284.6 eV, and background subtraction was performed using the Shirley method. Peak fitting was conducted using a GL (30) line shape.

Compared with the SDS-free plating system, pronounced differences were observed upon the addition of SDS to the plating bath, particularly in the high-resolution Ni and S spectra. Specifically, in the Ni 2p spectra, the main peaks located at ∼852 and 869 eV correspond to the Ni 2p_3/2_ and Ni 2p_1/2_ components, respectively, which are attributed to the Ni and Ni–P bonding states in the Ni–P alloy. Comparison of Figures 4C,D indicates that the sample plated in the presence of SDS exhibits relatively broader main peaks compared with the sample plated in the absence of this surfactant, implying that SDS modified the surface chemical environment of Ni. The peaks observed at ∼855 and 873 eV correspond to the satellite features of Ni 2p_3/2_ and Ni 2p_1/2_, respectively (Biggio et al., 2024; Farhan et al., 2022; Oguocha et al., 2010). These satellite peaks are commonly attributed to oxidation-related Ni states and have also been associated with nickel phosphate species in studies of electroless Ni–P plating (Biggio et al., 2024). Additional satellite features are further observed at ∼860 and 879 eV.

In the high-resolution S 2p spectra, a clear S 2p3/2–2p1/2 doublet structure is observed, arising from spin–orbit splitting. The S 2p3/2 peak located at ∼161.5–162.1 eV can be cautiously assigned to possible Ni–S-related sulfur species, while the component at ∼168 eV is associated with oxidized (sulfate-type) sulfur species (Legrand et al., 1998; Wagner and NIST, 1991; Wang et al., 2017; Yang et al., 2022). For the sample prepared in the absence of SDS (Figure 4E), the intensity of the Ni–S-related S 2p component is approximately 2.6 times lower than that of the sample prepared with added SDS (Figure 4F), while the thiourea concentration was identical in both baths. In contrast, the oxidized sulfur component shows a relatively lower intensity in the sample prepared in the presence of SDS. Given the constant thiourea concentration across both samples, this systematic increase in the Ni–S-related component and corresponding decrease in the oxidized sulfur component with SDS addition suggest that SDS contributes to a shift in the relative distribution of sulfur-containing surface species toward Ni–S-related states. The atomic compositions obtained from XPS analysis are summarized in Table 1. As shown in Table 1, the SDS-containing sample showed an increased relative Ni 2p concentration but decreased Na 1s and P 2p concentrations. Since XPS provides relative surface composition within a limited analysis depth, these values do not necessarily represent bulk compositional changes in the entire Ni–P layer. Although SDS contains sodium, it dissociates in the aqueous plating bath, and Na-containing species may remain in the solution phase or be removed during rinsing. The lower Na 1s and P 2p signals are therefore more cautiously interpreted as changes in the relative outermost surface composition rather than changes in the bulk composition of the Ni–P layer.

**TABLE 1:** Surface atomic concentrations obtained from XPS analysis of the samples prepared using Bath A and Bath B.

Element	Atomic concentration (%)	
	Bath A	Bath B
O 1s	43.13	34.32
C 1s	25.76	33.45
Ni 2p	20.46	27.83
Na 1s	5.58	1.07
P 2p	2.62	1.08
S 2p	2.45	2.26

**FIGURE 5:**
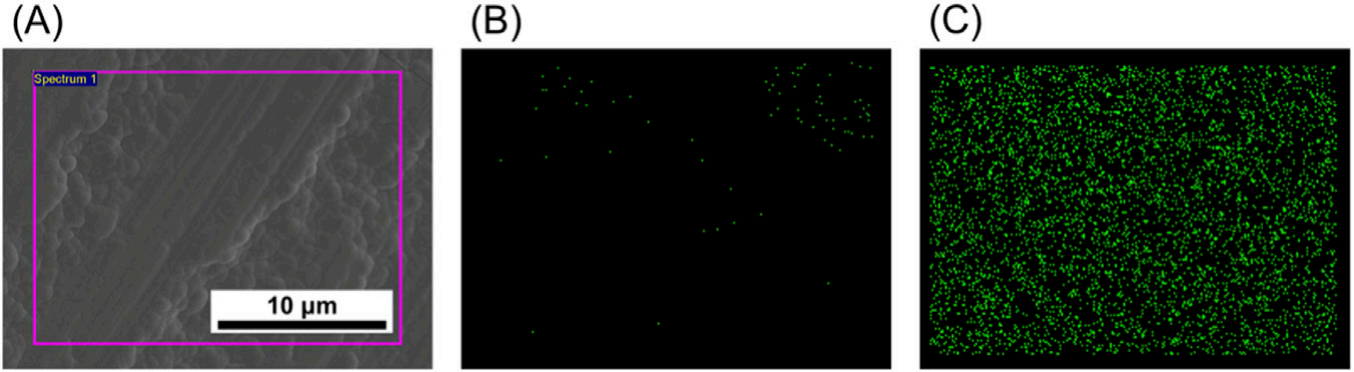
SEM image and SEM mapping results of the TE element surface before and after Pd activation: **(A)** SEM image of the bare TE element surface, **(B)** Pd mapping image of the TE element surface before Pd activation, and **(C)** Pd mapping image of the TE element surface after Pd activation.

To confirm the formation and distribution of a catalytic Pd layer during activation, FE-SEM and EDX were performed. Figure 5A presents the SEM surface image of the bare TE element substrate prior to activation, while the Pd elemental mapping images recorded before and after Pd activation are shown in Figures 5B,C, respectively. No Pd signal was detected by EDX before activation, indicating that no catalytically active Pd^0^ species were present on the substrate surface at this point.

Figure 5C shows the Pd elemental mapping image obtained after the activation process, revealing a Pd concentration of ∼36 wt%. This result indicates successful deposition and strong adherence of the Pd layer to the TE element substrate. The relatively high Pd content suggests the formation of a dense and well-distributed catalytic layer rather than isolated or sparsely distributed particles. Moreover, the uniform spatial distribution of Pd across the TE element surface provides a homogeneous population of catalytic sites, which is essential for achieving consistent nucleation and growth during electroless Ni–P plating.

**FIGURE 6:**
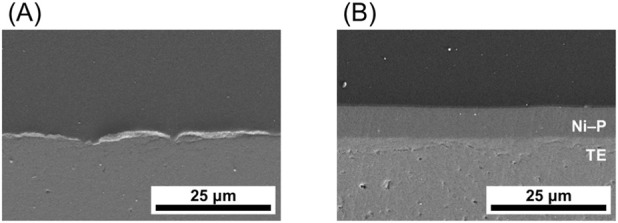
Cross-sectional SEM images of electroless Ni–P-plated TE elements prepared using **(A)** Bath A (without SDS) and **(B)** Bath B (with SDS).

Following confirmation of successful Pd activation, electroless Ni–P plating was performed to examine the effects of SDS on the plating behavior and resulting film morphology. To examine the effects of SDS on the plating solution/TE element interfacial characteristics and plating morphology during the electroless plating process, electroless Ni–P plating was conducted both in the presence and absence of SDS at a fixed plating time of 20 min. Subsequently, the plating morphology was characterized using cross-sectional SEM imaging. In the absence of SDS (Bath A), the Ni–P layer was extremely thin and non-uniform, with thicknesses of 0.63–1.66 μm (Figure 6A). Additionally, the plated surface exhibited a rough and irregular morphology. In contrast, electroless plating in the presence of SDS (Bath B) resulted in a uniform Ni–P plating layer with an average thickness of 5 μm (Figure 6B), which exhibited a smooth and continuous surface morphology.

**FIGURE 7:**
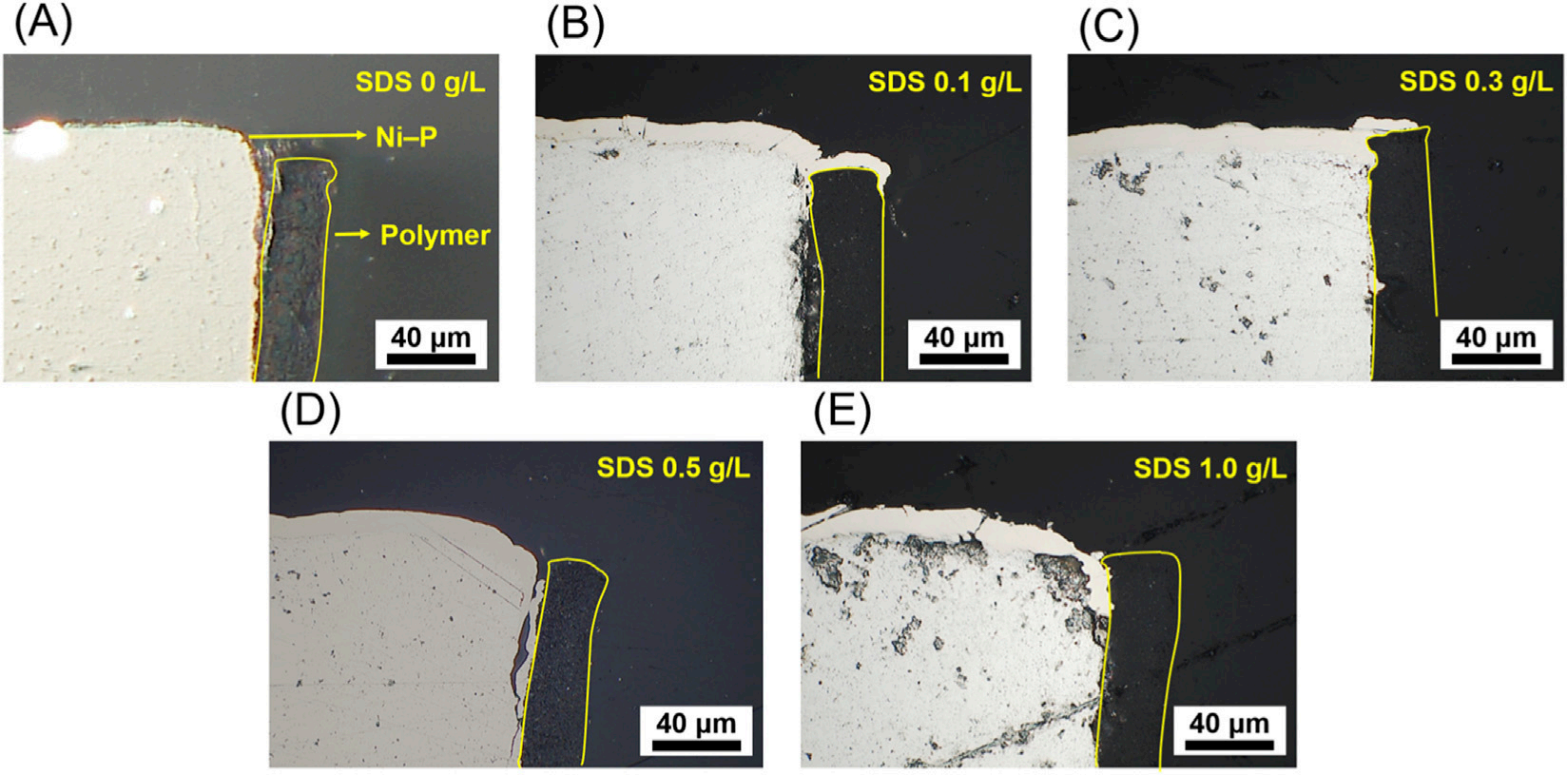
Cross-sectional OM images of electroless Ni–P-plated TE elements prepared with different SDS concentrations: **(A)** 0 g/L, **(B)** 0.1 g/L, **(C)** 0.3 g/L, **(D)** 0.5 g/L, and **(E)** 1.0 g/L.

Figure 7 shows cross-sectional optical microscopy (OM) images of the Ni–P-plated TE elements prepared with SDS concentrations of 0, 0.1, 0.3, 0.5, and 1.0 g/L. In the absence of SDS (Figure 7A), a thin, non-uniform, and discontinuous Ni–P layer was observed on the TE element surface, consistent with the poor wetting behavior discussed in relation to Figure 6A. With the addition of SDS, the formation of the Ni–P plating layer on the TE element surface improved under otherwise identical plating conditions. However, at SDS concentrations of 0.1 and 0.3 g/L (Figures 7B,C), Ni–P deposition was observed not only on the TE element region but also, to a limited extent, in the adjacent polymer region, indicating that plating selectivity was not yet sufficiently achieved at these concentrations. At an SDS concentration of 0.5 g/L (Figure 7D), selective Ni–P plating was successfully achieved, with a continuous Ni–P layer formed on the TE element region while deposition on the polymer region remained effectively suppressed. In contrast, at an SDS concentration of 1.0 g/L (Figure 7E), the Ni–P-plated region extended further toward the polymer region, indicating that excessive SDS addition deteriorated plating selectivity. These results indicate that SDS concentration governs both the extent of Ni–P coverage on the TE element surface and the degree of plating selectivity relative to the polymer region, and that an intermediate SDS concentration is required to simultaneously achieve adequate TE element coverage and effective suppression of polymer-region deposition. On this basis, 0.5 g/L SDS was selected as the representative concentration for the subsequent characterization presented in this study.

**FIGURE 8:**
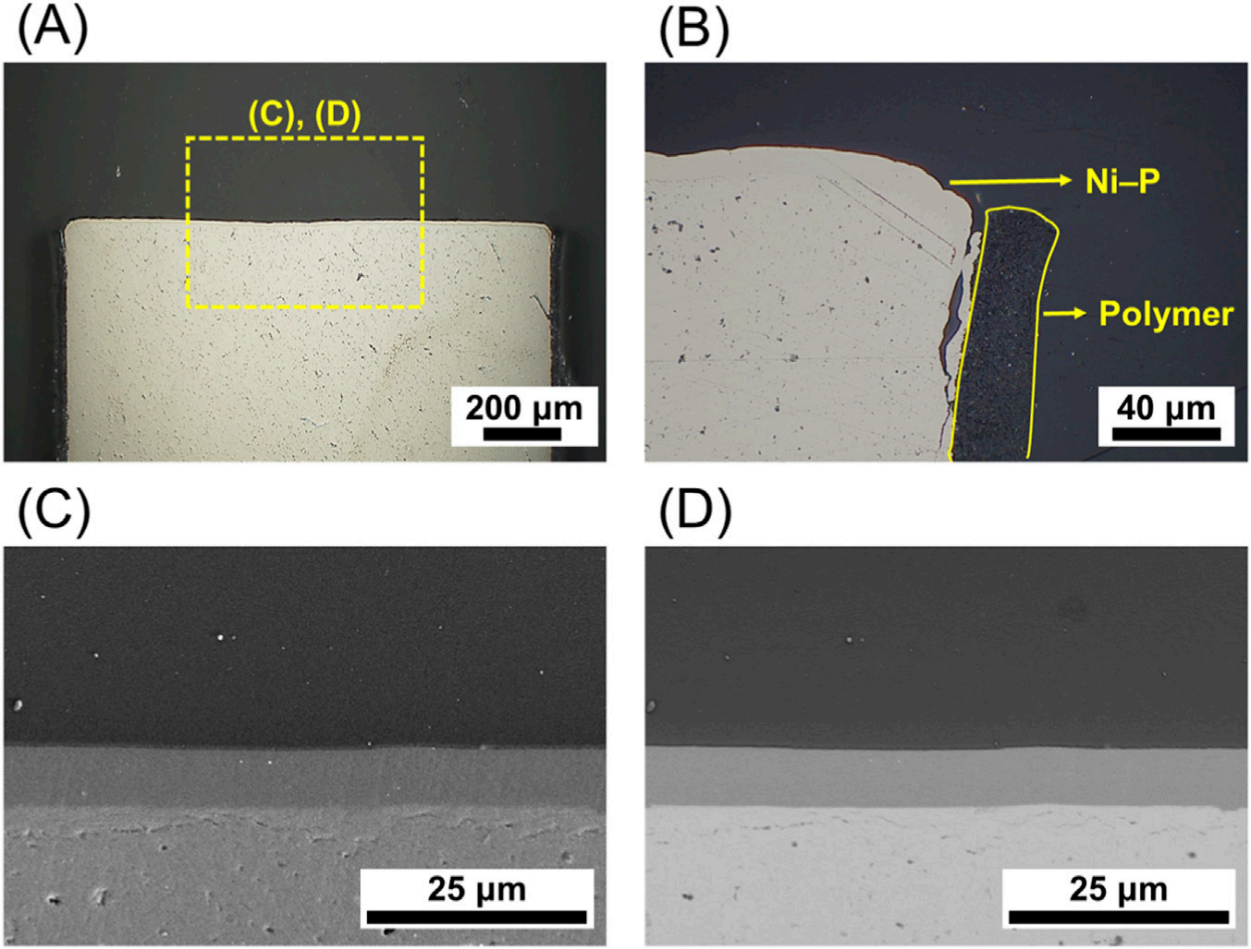
Cross-sectional OM and SEM images of electroless Ni–P-plated TE elements prepared using Bath B: **(A)** full OM image, **(B)** OM image of the polymer section, **(C)** SEM image, and **(D)** BSE image.

Figures 8A,B show the OM images of the cross-sections of the TE elements following electroless Ni–P plating in the presence of SDS (Bath B). The magnified OM image in Figure 8B clearly demonstrates selective electroless Ni–P plating on the TE element region, whereas no plating is observed in the polymer region. In addition, the SEM and backscattered electron (BSE) images shown in Figures 8C,D confirm the formation of a uniform and continuous Ni–P plating layer in the presence of SDS.

To evaluate the performance of the Ni–P diffusion barrier layer formed using SDS-containing Bath B, a Cu layer was deposited through electroplating to function as a solder layer. To investigate the potential for interdiffusion and intermetallic compound formation at elevated temperatures, the samples were subjected to heat treatment at 100 °C and 200 °C.

**FIGURE 9:**
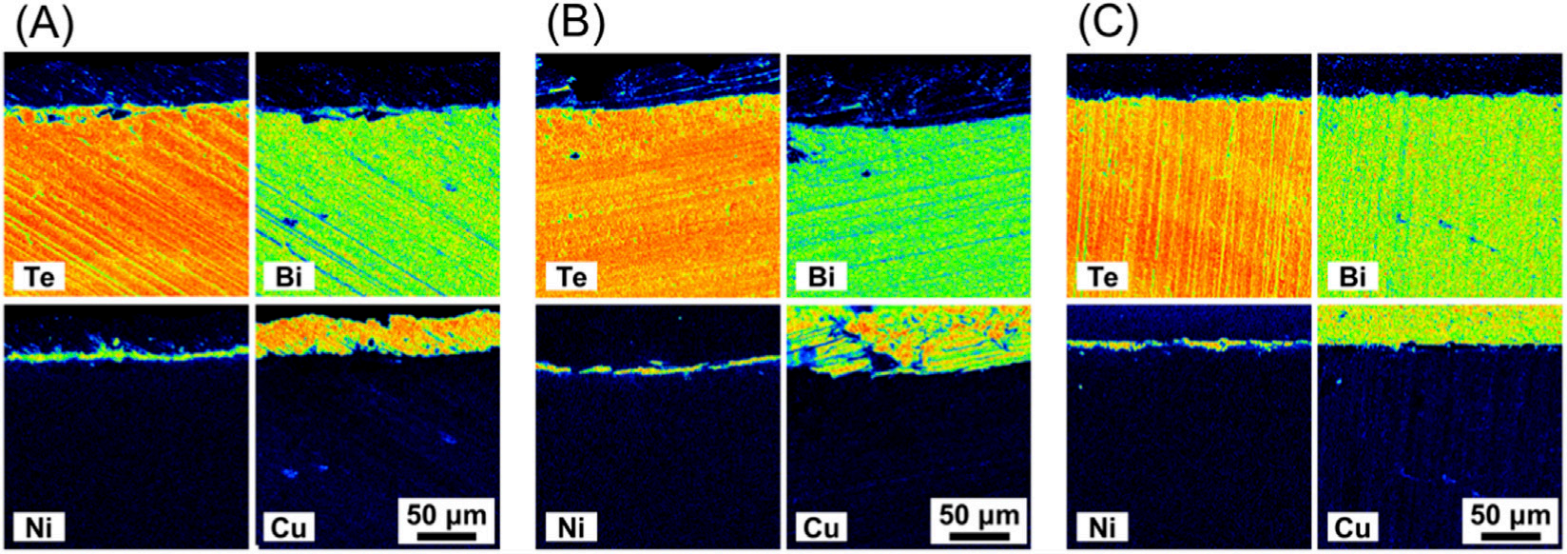
Cross-sectional EPMA elemental analysis of the Cu/Ni–P-plated TE elements: **(A)** as-deposited specimen and specimens after heat treatment at **(B)** 100 °C and **(C)** 200 °C.

The microstructural changes of the Cu/Ni–P-plated TE elements, along with those of the heat-treated samples, were analyzed and compared using cross-sectional EPMA analysis. Figure 9A shows the EPMA results for the as-deposited TE element, where the presence of Cu and Ni is confirmed. Figures 9B,C confirm that Cu did not diffuse into the Ni–P layer following heat treatment at either 100 or 200 °C, revealing the effectiveness of the Ni–P diffusion barrier layer.

**FIGURE 10:**
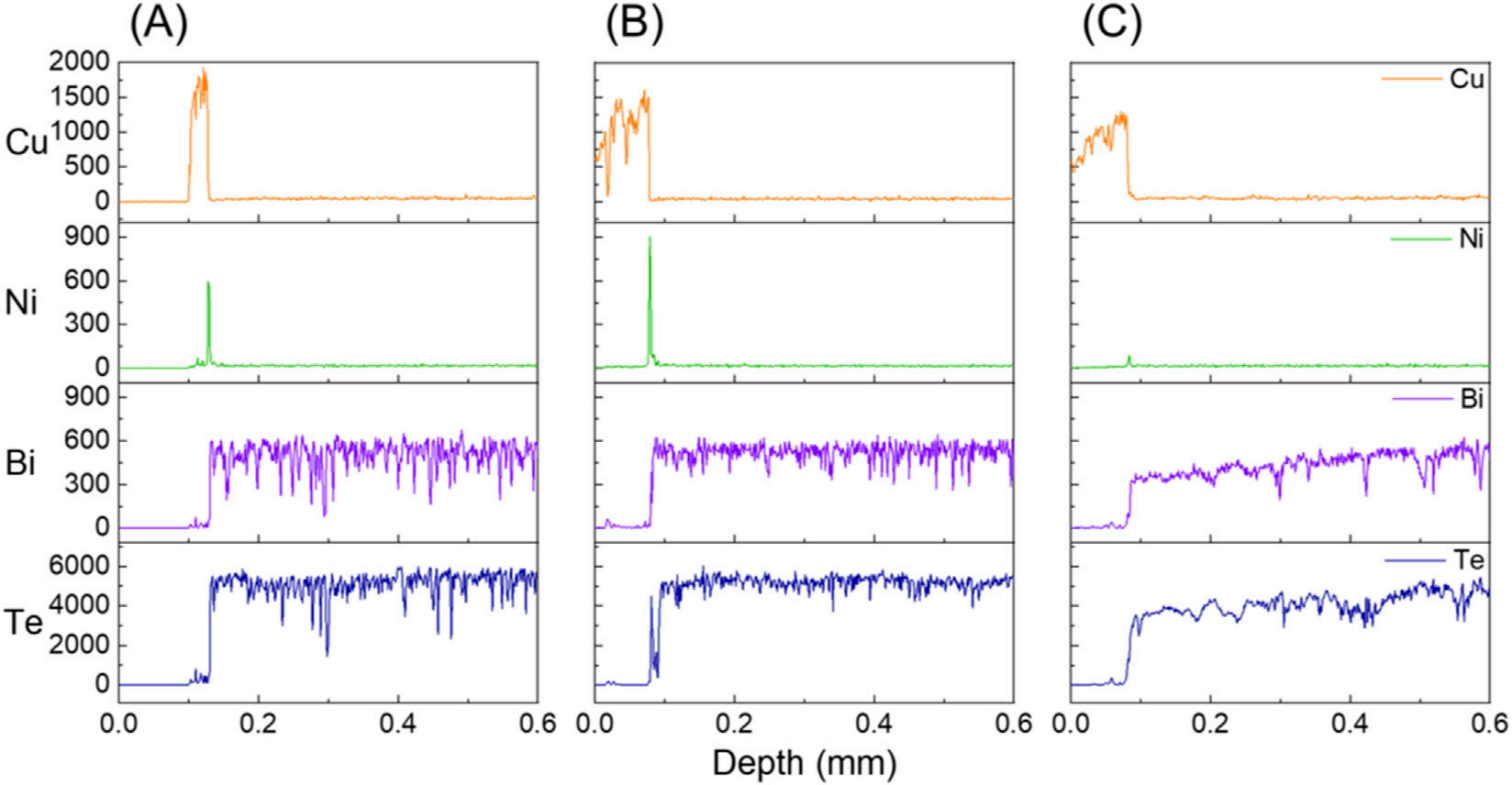
Cross-sectional EPMA line-scan analysis of the Cu/Ni–P-plated TE elements: **(A)** as-deposited specimen and specimens after heat treatment at **(B)** 100 °C and **(C)** 200 °C.

Figure 10 presents the EPMA line-scan analyses of the elemental distribution across various depths of the TE elements, which were used to evaluate the effectiveness of the Ni–P diffusion barrier layer by identifying possible Cu diffusion. Figure 10A shows the EPMA line-scan profile of the as-deposited Cu/Ni–P TE element, and Figures 10B,C show the corresponding profiles after heat treatment at 100 °C and 200 °C, respectively. In all cases, no Cu diffusion occurred beyond the Ni–P barrier layer, with Cu remaining undetected in the Bi and Te regions of the TE material. Notably, no intermetallic compound formation was observed. These results confirm that the Ni–P barrier layer effectively suppresses Cu diffusion and prevents interfacial reactions with the TE element, demonstrating its suitability as a diffusion barrier.

Since Bi–Sb–Te-based thermoelectric devices operate over a relatively low-temperature regime, with optimal thermoelectric performance typically observed at ∼100 °C, the diffusion stability at 100 °C is particularly important from the viewpoint of practical applications (Goldsmid, 2010; Snyder and Toberer, 2008).

Notably, although variations in the absolute intensities of Cu and Ni were observed in the EPMA line-scan profiles, these differences do not indicate interdiffusion. For instance, the non-uniform Cu thickness is attributed to limitations in electrical contact during electroplating on the small TE elements. Additionally, the variation in Ni signal intensity arises from the thin electroless Ni–P layer and matrix effects associated with the adjacent Cu and thermoelectric materials, which influence the effective interaction volume during EPMA analysis. Importantly, the Ni layer remains continuous and well-defined at the interface, and no Cu signal is detected within the thermoelectric region, further confirming the effectiveness of the Ni–P layer as a diffusion barrier.

## 4 Discussion

Improved wettability plays a critical role in enhancing the uniformity and adhesion of electrochemical deposits while enabling selective deposition (Bae et al., 2019; Elansezhian et al., 2008; Farzaneh et al., 2010; Wang et al., 2019). Specifically, a reduced contact angle allows the plating solution to cover the substrate more evenly, minimizing localized concentration gradients and suppressing the formation of voids or interfacial defects. This pronounced reduction in contact angle can be attributed to the surfactant behavior of SDS, which contains a hydrophilic sulfate head group (–OSO_3_^−^) and a hydrophobic alkyl tail. When SDS is introduced into the plating bath, it can reduce the surface tension of the aqueous solution and improve solid–liquid interfacial contact (Farzaneh et al., 2010; Ivanova and Starov, 2011). The enhanced spreading on the Bi–Sb–Te TE element surface is directly supported by the contact angle results, where SDS addition reduced the contact angle from 50° to 9°. This behavior can be understood through Young’s equation,

$$\cos\theta =\left(\gamma \text{SV}-\gamma \text{SL}\right)\;/\;\gamma \text{LV}$$

where θ is the equilibrium contact angle, γSV is the interfacial energy between the solid surface and the surrounding vapor (i.e., the surface energy of the solid), γSL is the interfacial energy between the solid surface and the plating solution, and γLV is the surface tension of the plating solution at the liquid–vapor interface (Roura and Fort, 2004). According to this relation, the marked decrease in contact angle reflects the combined effect of the intrinsically higher surface energy (γSV) of the activated TE element surface and the SDS-induced reduction in the surface tension of the plating bath (γLV). The resulting near-complete wetting (θ ≈ 9°) allows the plating bath to cover the TE element surface more uniformly, thereby facilitating homogeneous Ni–P nucleation during the initial stage of electroless deposition. This contrast in wetting behavior between the TE element and polymer regions can be attributed to the substantially lower surface energy (γSV) and chemically inert nature of the polymer substrate. Because the solid–liquid interaction term (γSV − γSL) for the polymer surface is intrinsically small, the same reduction in γLV afforded by SDS is insufficient to achieve comparably favorable wetting there: the polymer surface maintained a relatively high contact angle even after SDS addition, indicating limited spreading of the plating solution on the polymer region. As a result, the plating solution preferentially wetted the higher-energy TE element surface, while spreading on the low-energy polymer surface remained restricted, providing the physicochemical basis for the observed selectivity of electroless Ni–P deposition. This wettability contrast between the TE element and polymer regions is a key factor governing selective electroless deposition, since the well-wetted TE element surface supports uniform solution coverage and reaction-site accessibility, whereas the poorly wetted polymer region restricts spreading and suppresses Ni–P deposition (Huang et al., 2018; Ivanova and Starov, 2011; Meconi et al., 2016; Wang et al., 2019; Wang et al., 2025). Consequently, SDS contributes to the formation of a dense and uniform Ni–P coating while enabling selective deposition.

Notably, the electroless plating bath employed in this study contains thiourea as well as SDS, both of which are sulfur-containing additives, making it difficult to unambiguously assign a single sulfur source to the observed Ni–S-related species. However, because the thiourea concentration was held constant across all samples, any SDS-dependent variation in the sulfur-related surface chemistry can be attributed, at least in part, to SDS rather than to thiourea alone. Consistent with this, the relative intensity of the Ni–S-related S 2p peak, together with the corresponding changes in Ni 2p chemical states, varied systematically with SDS concentration. This trend suggests that SDS addition contributes to the observed changes in sulfur-related surface chemical states, although, based on the present XPS results alone, the individual contributions of SDS and thiourea to the Ni–S-related signal cannot be fully deconvoluted.

In addition to enhancing plating uniformity and surface coverage, the sulfur-related surface species observed in the SDS-containing bath may have implications for the electronic properties of the Ni–P/TE element interface. In p-type Bi–Sb–Te-based TE elements, hole transport is the dominant conduction mechanism. According to the Schottky–Mott model, the hole barrier height at a metal/p-type semiconductor interface is given by φB,p = Eg − (φM − χS), where φM is the metal work function, χS is the electron affinity of the semiconductor, and, Eg is its band gap; an increase in φM therefore reduces φB,p and, correspondingly, the contact resistance for hole injection (Meng and Li, 2020; Sze and Ng, 2007). A work function mismatch that increases this barrier can thus increase contact resistance (Meng et al., 2024; Çankaya and Uçar, 2004), which is detrimental to TE device performance because it degrades charge-carrier transport and reduces power output and conversion efficiency (Li et al., 2015; Xia et al., 2014). Sulfur-containing surface species such as NiS have been reported to modify interfacial electronic structure and charge-transfer behavior in other metal–semiconductor systems—for example, by acting as an electron-trapping co-catalyst at NiS/TiO_2_ Schottky-type junctions (Qiao et al., 2019) and by contributing to built-in-field-driven charge separation at transition-metal sulfide heterojunctions (Zhang et al., 2023). These reports do not directly establish the work function of NiS relative to metallic Ni (whose clean-surface work function has been compiled by Derry et al. (2015), nor do they address a Ni–P/TE element interface; the discussion here is therefore an extrapolation from these analogous, non-TE systems rather than direct evidence for the present interface. On this basis, we consider it possible that the sulfur-related chemical states observed in the SDS-containing sample could, in principle, elevate the effective work function of the Ni-based contact layer and thereby reduce φB,p at the Ni–P/TE element interface. This interpretation is subject to several limitations. The XPS analysis used to identify these sulfur-related species is inherently surface-sensitive and may not fully represent the bulk chemical composition of the Ni–P layer. More importantly, the proposed influence of sulfur-related surface species on the hole injection barrier is based on an extrapolation from analogous, non-TE sulfide systems rather than direct evidence for the Ni–P/TE element interface studied here, as the work function and barrier height of this specific interface were not directly measured. Quantitative evaluation of contact resistance was also not performed in the present work. Accordingly, this proposed mechanism should be regarded as a plausible interpretation rather than an experimentally confirmed effect, and future studies incorporating direct work function measurements, band-alignment evaluation, and contact-resistance measurements will be required to validate this possibility.

Following the surface chemical analysis, the role of Pd activation in initiating electroless Ni–P deposition was considered. During the activation process, Pd ions introduced from the activation solution are chemically reduced on the substrate surface, producing metallic Pd^0^ species. This activation mechanism is widely recognized as a critical step in electroless plating, as it generates Pd^0^ nanoparticles that function as catalytic centers for subsequent metal deposition. Specifically, these Pd^0^ sites facilitate electron transfer at the surface, reducing Ni^2+^ ions and triggering the autocatalytic growth of the Ni–P layer (Cui et al., 2011; D’Amico et al., 1971; Loto, 2016; Wei and Roper, 2014). The uniform distribution of Pd observed after activation therefore supports homogeneous Ni–P nucleation and contributes to improved plating reproducibility, thickness uniformity, and overall coating quality.

Since electroless Ni–P plating proceeds via a surface-catalyzed redox reaction, the absence of Pd^0^ before activation implies that no catalytic nucleation sites were available to initiate the reduction of Ni^2+^ ions, thereby preventing the onset of electroless plating. This confirms that the presence of Pd^0^ on the substrate surface is a critical prerequisite for initiating and sustaining uniform electroless Ni–P plating (D’Amico et al., 1971).

Following the catalytic activation step, the comparison of Ni–P deposition behavior with and without SDS, as well as at different SDS concentrations, demonstrates that SDS plays an important role in determining both the quality of selective plating and the uniformity of the Ni–P coating. This improvement in plating uniformity is consistent with the significantly reduced contact angle observed upon SDS addition (Figure 3), which enhanced solution spreading and interfacial contact during plating (Ivanova and Starov, 2011). The smoother and more uniform surface observed after plating with SDS can also be attributed to the interfacial and colloidal stabilization effects of SDS. As an anionic surfactant, SDS can interact with positively charged Ni^2+^ ions in the plating bath, suppressing excessive aggregation of Ni-containing species and localized overgrowth during deposition. Similar effects of SDS and other surfactants on grain refinement, particle dispersion, and deposit uniformity have been reported in previous electrochemical deposition studies (Elansezhian et al., 2009; Farzaneh et al., 2010; Guo et al., 2008). Consistent with these reports, SDS addition in this study contributed to the formation of a denser, smoother, and more uniform Ni–P coating. Moreover, the SDS concentration strongly influenced the plating selectivity at the TE element/polymer boundary. The optimized SDS concentration of 0.5 g/L promoted continuous Ni–P deposition on the TE element region while suppressing unwanted deposition on the adjacent polymer region, whereas excessive SDS addition caused the plated region to extend toward the polymer area.

The EPMA results further confirm that the SDS-assisted electroless Ni–P layer functioned effectively as a Cu diffusion barrier. Even after heat treatment at 100 °C and 200 °C, Cu remained confined to the outer Cu layer and was not detected within the Bi–Sb–Te TE element region, indicating that the continuous Ni–P layer suppressed Cu migration and interfacial reaction. The stability observed at 100 °C is particularly relevant to the peak-performance temperature range of Bi–Sb–Te-based TE devices (approximately 25 °C–125 °C), indicating that the Ni–P barrier layer maintains its diffusion-blocking capability under the thermal conditions in which the device operates at its highest efficiency. The result at 200 °C, well beyond this range, further suggests that the barrier layer retains a degree of diffusion-blocking capability even under more severe, thermally accelerated exposure. Therefore, the uniform Ni–P layer formed with SDS not only improved coating morphology and selectivity but also contributed to the thermal stability of the metallized TE structure.

## 5 Conclusion

In this study, selective electroless Ni–P plating was successfully applied to polymer-coated cylindrical TE elements to prevent deposition on adjacent polymer regions, thereby preserving the temperature gradient critical for device efficiency. The incorporation of SDS into the plating bath significantly improved the wettability and uniformity of the Ni–P coating formed on the TE element surface. Specifically, the marked contrast in contact angles between the TE element and polymer substrates enabled selective deposition, where uniform plating occurred on the TE element and was effectively suppressed on the polymer due to reduced wettability. This study is distinct from previous reports on SDS-modified electroless Ni–P plating, which have primarily addressed deposit-wide roughness or hardness on single, flat, conductive substrates, in that selective electroless Ni–P plating was directly demonstrated on polymer-coated cylindrical TE elements for Cu diffusion barrier formation. In addition, the effects of SDS addition were systematically examined in relation to plating selectivity, surface and interfacial chemical states, and Cu diffusion barrier performance. Additionally, the electroless Ni–P layer functioned as an effective diffusion barrier, suppressing Cu diffusion from the solder and inhibiting the formation of intermetallic compounds that typically degrade electrical and mechanical properties. Consequently, the Ni–P barrier enhanced the long-term stability and reliability of the TE module. Overall, these findings demonstrate that SDS-assisted selective electroless Ni–P plating represents a viable and scalable strategy for improving the performance and durability of thermoelectric devices, offering valuable implications for energy harvesting and thermal management applications. The addition of SDS thus offers a simple and cost-effective means of inducing wettability contrast between the dissimilar regions of a hybrid TE element substrate, enabling selective Ni–P deposition and enhanced interfacial reliability without introducing additional fabrication steps. Owing to its simplicity and compatibility with large-scale production, this process presents a highly practical solution for efficient thermoelectric device integration. Future work will focus on directly evaluating the work function, barrier height, and contact resistance.

## Data Availability

The original contributions presented in the study are included in the article/supplementary material, further inquiries can be directed to the corresponding author.
